# Supplementary material for: Multiplexed CRISPR/CAS9‐mediated engineering of pre‐clinical mouse models bearing native human B cell receptors
Source: EMBO J. 2020 Dec 1;40(2):e105926. doi: 10.15252/embj.2020105926 (PMC7809789; doi:10.15252/embj.2020105926)
Supplement: Supplementary file 1 — Appendix [file EMBJ-40-e105926-s001.docx]

**Appendix**

**Multiplexed CRISPR/CAS9-Mediated Engineering of Pre-clinical Mouse Models Bearing Native Human B Cell Receptors**

Xuesong Wang^1,†^, Rashmi Ray^1,†^, Sven Kratochvil^1^, Eleonora Melzi^1^, Ying-Cing Lin^1^, Sophie Giguere^1^, Liling Xu^1^, John Warner^1^, Diane Cheon^1^, Alessia Liguori^4,5,6^, Bettina Groschel^4,5,6^, Nicole Phelps^4,5,6^, Yumiko Adachi^4,5,6^, Ryan Tingle^4,5,6^, Lin Wu^7^, Shane Crotty^6,8,9^, Kathrin H. Kirsch^1^, Usha Nair^1^, William R. Schief ^1,4,5,6^,*, and Facundo D. Batista ^1,2,3^*

**Affiliations:**

^1^The Ragon Institute of Massachusetts General Hospital, Massachusetts Institute of Technology and Harvard University, Cambridge, MA 02139, USA.

^2^ Department of Immunology, Harvard Medical School.

^3^Department of Microbiology, Harvard Medical School.

^4^Department of Immunology and Microbiology, The Scripps Research Institute, La Jolla, CA 92037, USA.

^5^IAVI Neutralizing Antibody Center, The Scripps Research Institute, La Jolla, CA 92037, USA.

^6^Consortium for HIV/AIDS Vaccine Development (CHAVD), The Scripps Research Institute, La Jolla, CA 92037, USA.

^7^Genome Modification Facility, Department of Molecular and Cellular Biology, Harvard University, Cambridge, MA, USA.

^8^Center for Infectious Disease and Vaccine Research, La Jolla Institute for Immunology (LJI), La Jolla, CA 92037, USA.

^9^Department of Medicine, University of California, San Diego, La Jolla, CA 92037, USA

†Contributed equally

*Correspondence to: [fbatista1@mgh.harvard.edu](mailto:fbatista1@mgh.harvard.edu), schief@scripps.edu

**This Appendix includes:**

**• Appendix Figure S1-S10**

**•** **Supplementary Methods**

**• Appendix Table S1-S7**

**Appendix Figure S1.** **Generation and detection of PGT121 κ and CLK double KI mice**

(A) Table shows the genotyping result and the frequency of human PGT121 κ mice generated after CRISPR injection.

(B) Analysis of human PGT121 κ associated with murine heavy chain. Left column shows the sorted B cell frequency. Each pie represents the frequency of PGT121 human κ paired with mouse HC in each mouse. Right bar represents the frequency of mouse HC families which were paired with PGT 121 human κ.

(C) Strategy for the insertion of pre-rearranged CLK VJ and VDJ into the mouse native locus. In order to generate the double KI CLK mice, fertilized mouse oocytes were microinjected with two donor plasmids each bearing CLK gl H and κ pre-rearranged sequences and the relevant 5’ and 3’ homology arms, mouse VHJ558 or Vκ4-53 promoter (P) and corresponding leader regions, four sgRNAs—with two sgRNAs (H1, H8 or L18, L11) targeting each H or κ locus, and Cas9 as Figure 1A and our previous paper described (Lin et al., 2018). Red rectangles show the rearranged CLK VJ (left) or CLK VDJ (right). “T” represents TaqMan probe. WT probes were used for the detection of WT allele, leader probes were used for the detection of the 5’end of the insertion, the specific probes were used for the detection of pre-arranged VJ or VDJ insertion for each line respectively (probe sequences, see Table S1).

**Appendix Figure S2. Characterization of B-lymphocyte development in the bone marrow of CLK21, CLK09 and CLK19 human BCR knock-in mice.**

A representative F1 breeder from each line was tested for B lymphocyte development.

(A) Bone marrow cells from WT, CLK21, CLK09 and CLK19 human BCR knock-in mice were analyzed by flow cytometry using the gating strategy shown on the left. B-cell progenitors (B220^+^) were divided into immature (CD43^+^) and mature (CD43^-^) cells on the basis of CD43 expression.

(B) Early (CD43^+^) B-cell progenitors were subdivided according to CD24 and BP-1 expression into Hardy populations A (CD24^-^BP-1^-^), B (CD24^+^BP-1^-^), and C (CD24^+^BP1^+^).

(C) Late (CD43^-^) B-cell progenitors were subdivided according to IgM and IgD expression into Hardy populations D (IgM^-^IgD^-^), E (IgM^+^IgD^int^), and F (IgM^+^IgD^+^).

**Appendix Figure S3. Characterization of B- and T-lymphocyte development in spleen of CLK21, CLK09 and CLK19** **human BCR knock-in mice.**

A representative F1 breeder from each line was tested for B and T lymphocytes development.

Spleens from WT, CLK21, CLK09 and CLK19 human BCR knock-in mice were analyzed by flow cytometry. (A, B) Identification of B cells (B220^+^TCRb^-^) and T cells (B220^-^TCRb^+^). T cells were subdivided into CD4 (CD4^+^CD8^-^) and CD8 (CD4^-^CD8^+^) T cells.

(C, D) B cells were divided on the basis of CD21, CD23, and CD24 expression into T0/T1 cells (CD2^-^CD24^hi^), follicular B cells (CD21^lo^CD24^lo^), T2 cells (CD21^hi^CD24^hi^CD23^-^), marginal zone B (MZB) cells (CD21^hi^CD24^hi^CD23^+^).

**Appendix Figure S4. Detection of mRNA production of** **Ig M and Ig** κ **in CLK21 human BCR knock-in mice.**

(A) mRNA production level of human Ig M and Ig κ in CLK21 KI mice. mRNA was isolated from the blood of 8-week old KI mice, RT-PCR was used to detect the mRNA production level. Upper table shows the genotyping results of F0 mice and lower figure shows the RT-PCR result of Ig M and Ig κ mRNA production level in CLK21 KI mice.

(B) Bulk mRNA next generation sequencing of BCRs from WT, *H^CLK21/WT^κ^WT/WT^*, *H^WT/WT^κ^CLK21/WT^* and *H^CLK21/WT^κ^CLK21/WT^* KI mice. Light gray represents mouse Ig H or Ig κ repertoire, green represents CLK21 human Ig M or Ig κ repertoire. The percentage represents the CLK21 Ig M or Ig κ clonotype frequency in KI mouse.

**
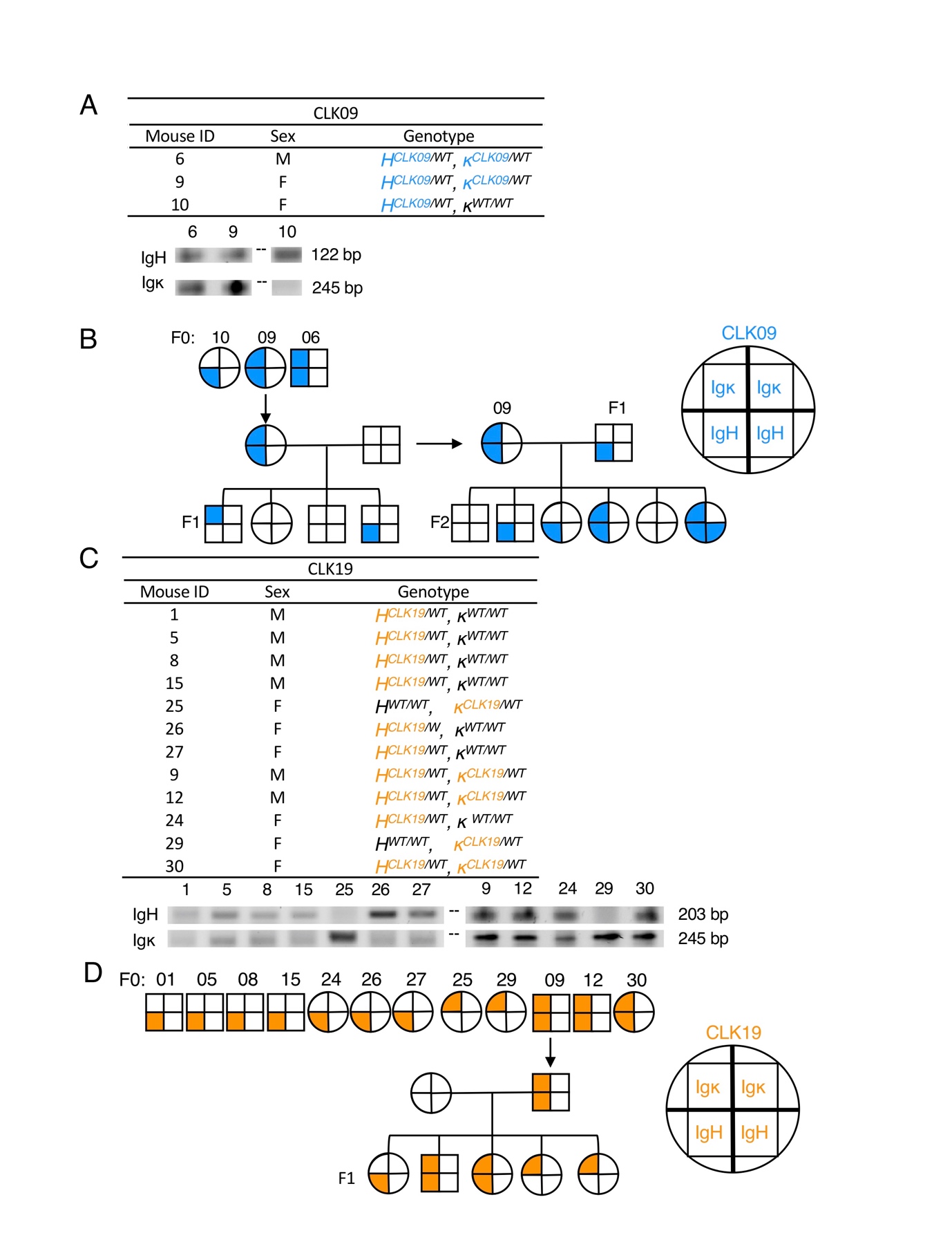
**

**Appendix Figure S5. Ig M and Ig** κ **mRNA detection and genetic map of CLK09 and CLK19.**

(A) mRNA production level of human Ig M and Ig κ in CLK09 KI mice. mRNA was isolated from the blood of 8-week old KI mice, RT-PCR was used to detect the mRNA production level. Upper table shows the genotyping results of F0 mice and lower figure shows the RT-PCR result of Ig M and Ig κ mRNA production level in CLK09 KI mice.

(B) CLK09 KI mice F0, F1 and F2 generations. Here squares and circles in the schematic represent male mice and circles represent as mentioned above. F0 generation mice genotyping results showing mouse 10 is *H^CLK09/WT^κ^WT/WT^*, 6 and 9 are *H^CLK09/WT^κ^CLK09/WT^*. Mouse 9 was crossed with WT to obtain four F1 progeny: one F1 mouse is *H^CLK09/WT^κ^WT/WT^*, one is *H^WT/WT^κ^CLK09/WT^*, and two are *H^WT/WT^ κ^WT/WT^*. Mouse 9 from F0 was crossed with *H^CLK09/WT^κ^WT/WT^* from F1 to obtain six F2 progeny: two F2 mice are *H^CLK09/WT^κ^WT/WT^*, one is *H^CLK09/WT^ κ^CLK09/WT^*, one is *H^CLK09/CLK09^κ^WT/WT^* and the other two mice are *H^WT/WT^ κ^WT/WT^*.

(C) mRNA production level of human Ig M and Ig κ in CLK19 KI mice. mRNA was isolated from the blood of 8-week old KI mice, RT-PCR was used to detect the mRNA production level. Upper table shows the genotyping results of F0 mice and lower figure shows the RT-PCR result of Ig M and Ig κ mRNA production level in CLK19 KI mice.

(D) CLK19 KI mice F0 and F1 generations. Here squares represent male mice and circles represent female mice. Upper halves of squares or circles represent Ig κ, and the lower halves represent Ig H, as shown in the schematic. F0 generation mice genotyping results showing mouse 1, 5, 8, 15, 24, 26 and 27 are *H^CLK19/WT^ κ^WT/WT^*, 25 and 29 are *H^WT/WT^ κ^CLK19/WT^*, and 9, 12 and 30 are *H^CLK19/WT^κ^CLK19/WT^*. Mouse 9 was crossed with WT to obtain five F1 progeny: one F1 mouse is *H^CLK19/WT^κ^WT/WT^*, two are *H^WT/WT^ κ^CLK19/WT^* and two are *H^CLK19/WT^ κ^CLK19/WT^*.

**Appendix Figure S6. GC frequency in CLK adoptively transferred mice with different precursor frequency over time.**

(A) GC frequency among total B cells on Day 8 in CLK adoptively transferred mice with different precursor frequency. Adoptively transferred mice with different precursor frequency were immunized with eOD-GT8 60 mer, on Day 8, the splenocytes were isolated and the GC frequency were detected by FACs with the marker SSL+B220+CD95+CD38-. Gated plots represent the GC frequency among B cells.

(B) Quantification of the frequency of GC B cells. Graph shows the quantification of GC frequency among B cells in three adoptively transferred mouse models after the immunization of eOD-GT8 60mer. X-axis represents the different precursor frequency group over time. Y-axis represents the percentage of GC B cells. Each circle represents one mouse. n=5 mice/group for CLK21 and CLK09, n=3 mice/group for CLK19. Bars indicate geometric mean and geometric SD from mice in each group. ns, no statistical significance.

**Appendix Figure S7. Class switch Recombination in GC B cells**

(A-B) Immunohistochemistry of day 36 spleen sections for host mouse receiving 5x10^5^ isolated CD45.2 B cells from CLK19 (left) and CLK09 (right) KI mouse. Green, B220; Blue, CD3; White, CD45.2; Red, GL7.

(C) Flow cytometry of the frequency of class switch recombined (CSR) IgG1 induced by eOD-GT8 60mer among GC CD45.2 binders. Adoptively transferred mice with 1 in 10^4^ of precursor frequency were immunized with eOD-GT8 60mer. At day 36, the splenocytes were isolated to detect the frequency of class switch recombined IgG1 in GC CD45.2 binders with the markers as SSL+, B220+CD4-CD8-F4/80-Gr-1-CD95+CD38-CD45.2+eOD-GT8+IgM-IgG1+ by FACS.

(D) Quantification of frequency of CSR IgG1 B cells among GC CD45.2 binders. Each circle represents one mouse, n=5 mice/group for CLK21 and CLK09, n=3 mice/group for CLK19. Bars indicate geometric mean and geometric SD from mice in each group. *p < 0.05, ****p < 0.0001, ns, no statistical significance.

**Appendix Figure S8. Memory B cells elicited by eOD-GT8 60 mer at day 36 post immunization**

(A) Flow cytometry of memory B cell (MBC) response induced by eOD-GT8 60mer in CLK21, CLK09, CLK19 adoptively transferred mice (1 in 10^4^ precursor frequency) on day 36. The panel of flow cytometric plots were prior gated from SSL+B220+CD4-CD8-F4/80-Gr-1-.

(B) Class switch memory B cells (CSM) were gated as SSL+B220+CD4-CD8-F4/80-Gr-1-CD38+GL7-IgD^low^/^-^IgM-. Green, blue and orange dots represent eOD-GT8 specific CD45.2 CSM for CLK21, CLK09 and CLK19 respectively.

(C) Quantification of CD45.2 MBC frequency for CLK21, CLK09 and CLK19 between eOD-GT8 60mer and eOD-GT8KO 60mer immunized group. N=5 mice/group for CLK21 and CLK09, n=3 mice/group for CLK19. Bars indicate geometric mean and geometric SD from mice in each group. *p < 0.05, ***p < 0.001, ns, no statistical significance.

**Appendix Figure S9. Antibody evolution of CLK in adoptively transferred mice with 1 in 10^4^ precursor frequency**

(A) Gating strategy of sorted B cells. CLK21, CLK09 and CLK19 mice were adoptively transferred with 1 in 10^4^ precursor frequency and immunized with eOD-GT8 60mer, at day 8, 15 and 36, spleenocytes were isolated and sorted as figure shown, IgM-IgD- and IgG1+ eOD-GT8 specific B cells were sorted respectively and performed singe cell PCR for human IgG and IgKappa sequencing.

(B) Phylogenetic trees of CLK19 over time. These trees were generated for CLK19 day8 sequences, CLK19 day8+day15 sequences and CLK19 day8+day15+day36 sequences using paired aminoacidic sequences that were joined and aligned by MUSCLE (Price et al., 2010). Clonal lineage trees were generated using FastTree and a Jones Taylor Thornton model for AA evolution (Jones et al., 1992). The length of the branches reflects sequence distance (also see in Figure 7A).

(C) Mature bnAbs-type aa HC mutations in CLK19 B cells over time. The red diagonal line indicates a 100% efficiency of VRC01-class bnAb-type HC mutations. The black stair step indicates a calculated VH1-2 antigen-agnostic mutation distribution, which might include Ab structure stabilizing mutations. The same figure for CLK19 at day36 is also shown in Figure 7D.

(D) SHM of CLK19 are detectable in both IGHV and IGLV over time with the immunization of eOD-GT8 60 mer.

(E) The occurrence of silent and non-silent mutations is plotted for CLK21, CLK19 and CLK09 (extracted from the IMGT database). A high number of V region mutations is detectable in the respective heavy chains.

(F) Distribution of select CLK B cell HC aa mutations at day36.Composite data from all mice are shown in each panel. For each CLK line, n=2 mice.

**Appendix Figure S10. Antibody evolution of CLK in adoptively transferred mice with 3 in 10^6^ precursor frequency**

Adoptively CLK09 and CLK21 transferred mice (precursor frequency:3 in 10^6^) were immunized with eOD-GT8 60mer. eOD-GT8-specific splenic IgG1+ or IgM-IgD- B cells were single-cell sorted at day 36 post immunization for single-cell PCRs.

(A) Phylogenetic trees of CLK09 and CLK21. These trees were generated using paired aminoacidic sequences solely isolated at day 36. Single paired amino acid sequences were joined and aligned using MUSCLE (Price, Dehal et al., 2010). Clonal lineage trees were generated using FastTree and a Jones Taylor Thornton model for AA evolution (Jones, Taylor et al., 1992). The length of the branches reflects sequence distance.

(B) SHM are detectable in both IGHV and IGLV at day 36 post immunization with eOD-GT8 60mer.

(C) Hotspot analysis. The quality of mutations was assessed via hotspots analysis for both heavy (left) and light (right) antibody chains. Weblogos were generated via publicly available online tools (https://weblogo.berkeley.edu/logo.cgi).

(D) Mature bnAbs-like aa VH1-2 mutations in CLK B cells at day 36. The red diagonal line indicates a 100% efficiency of VRC01-class bnAb-type VH1-2 mutations. The black stair step indicates a calculated VH1-2 antigen-agnostic mutation distribution, which might include mutations that improve expression or stabilize Ab structure (Briney et al., 2016).

**Supplementary Methods**

**Reverse transcription polymerase chain reaction (RT-PCR) -** RNA from KI mice were isolated with RNeasy® Protect Animal Blood Kit (Qiagen), cDNA was prepared with 1 µg of RNA as templates using Maxima First Strand cDNA Synthesis Kit (Thermo Fisher Scientific). PCR reactions were performed in 10 µl reaction system with 2 µl of cDNA as templates, 0.5 µl of specific forward primers for IgM or IgKappa from CLK21, CLK09 and CLK19 (**shown as Table S6**), 0.5 µl of reverse primer for constant region of IgM or IgKappa (**shown as Table S6**), 5 µl of PrimeSTAR® HS DNA Polymerase premix (Takarabio). In a thermocycler, the PCR reaction was denatured at 98˚C for 30sec followed by 35 cyclers of 98˚C for 10 sec, 58˚C for 15 sec and 72˚C for 60 sec, ending at 4˚C for 10 min.

**Next generation sequencing -** In order to detect the human IgM or Ig Kappa frequency in KI mice, the whole blood was prepared for RNA isolation with RNeasy® Protect Animal Blood Kit (Qiagen). For RT reaction, 1µg ± 0.4 µg total RNA was denatured in a thermocycler with 1 µl dNTP (10 mM, Thermo Scientific, cat #R0192), 0.6 µl of 10 µM Oligo dT primers (5’-AAGCAGTGGTATCAACGCAGAGTACT(30)VN) and 0.1 µl RNase Inhibitor (40 U/μl, Clontech) in a total volume of 6 µL at 65 ˚C for 5 min and cooled to 4 ˚C immediately after denaturation. 6.2 µl of RT reaction buffer consisting of 2 µl 5X first-strand buffer (Invitrogen # 18080044) , 0.69 µl DTT (100 mM, Invitrogen, cat #Y00147), 1.38 µl Betaine (5 M, Sigma, cat #B0300-1VL), 0.25 µl MgCl2 (Invitrogen, cat #AM9530G) diluted to 200 mM, 0.3 µl RNase Inhibitor (40 U/μl, Takara, # 2313A), 1.0 µl of 20 µM Template Switch Oligo, (TSO, GTGACTGGAGTTCAGACGTGTGCTCTTCCGATCTrGrGrG) and 0.58 µl SuperScript III reverse transcriptase (200 U/μl, Invitrogen # 18080044) were added. The reaction mixture was incubated at 42 ˚C in a thermocycler for 90 minutes followed by 85 ˚C for 5 minutes. A bead cleanup of the cDNA at (1:1) ratio using SPRIselect beads (Beckman Cloulter, cat # B23318) was performed followed by 2X washes with 80% molecular grade ethanol. Ethanol washed mixture was dried for 3-5 minutes and resuspended in Buffer EB (Qiagen, cat #1014609). For PCR1, 5.75 µl of bead purified cDNA with 0.25 µl of TSO forward primer (5’-AGACGTGTGCTCTTCCGATC) and 0.25 µl of reverse primer of IgM (5’-TACACGACGCTCTTCCGATCTNNNNNNNAGGGGGAAGACATTTGGGAAGG) or Igkappa(5’TACACGACGCTCTTCCGATCTNNNNNNNACTGGATGGTGGGAAGATGGATACAG) were added in 12.5 µl of HotStart ReadyMix (Kapa-bio) reaction system. The reaction mixture was denatured in a thermocycler at 95 ˚C for 3 min followed by 18 cyclers of 98 ˚C for 15 sec, 65 ˚C for 20 sec and 72 ˚C for 60 sec, ending with a final extension at 72 ˚C for 5 min. For PCR2, 4 µl of IgKappa or 8 µl of IgM product from PCR1 as templates followed by mixture with 2 µl of Index primer (**Table S7**) in 50 of reaction system. The mixture was denatured in a thermocycler at 95 ˚C for 3 minutes followed by 13 cyclers of 98 ˚C -15 sec, 65 ˚C -20 sec and 72 ˚C-60 sec, ending with a final extension at 72 ˚C for 5 min. A bead cleanup using Agencourt AMPure XP beads (Beckman Coulter, cat #A63880) of final product by first using a (1:2) ratio followed by a (1:5) ratio was performed followed by 2X washes with 80% molecular grade ethanol. The mixture was dried for 3-5 minutes and resuspended in Buffer EB (Qiagen, cat #1014609). For sequencing, to attain full length BCR sequences all NGS libraries were sent to Genewiz to be sequenced on an Illumina MiSeq using MiSeq Reagent Kit v3. On average approximately one million reads were generated for each demultiplexed sample. Demultiplexing were performed on the MiSeq using the D7xx and D50x indices.

**Appendix** Table S1 TaqMan assay sequence for heavy chain and light chain of each line

| **Heavy Chain** | **Probe name** | **Sequences** |
| --- | --- | --- |
| WT | Ighj4-2 WT | FP—CCACTATTGTGATTACTATGCTATGGACTAC  RP—CCTGGAGAGGCCATTCTTACCT  Reporter—ACGGTGACTGAGGTTCC |
| Leader | Ighm-12 KO | FP—GAAGACAAACCCCACAGGCT  RP—GGACACATTAACCTCAGAGGATGAC  Reporter—TACGCGTGCTAGCCTC |
| CLK09 | HuIghV-CLK09 Tg | FP—GTGTATTACTGTGCGAGAGTCGAA  RP—TTGGCCCCAGATATCAAAAGTCTTT  Reporter—CCCGAACCATAACCC |
| CLK19 | Clk19-GL-HC Tg | FP—GCTGAGCAGGCTGAGATCTG  RP—GTCAAAGCGCCATCGATAACC  Reporter—CCAGGGCGGTAGCACA |
| CLK21 | HuCLK21 TG | FP—GCTGAGCAGGCTGAGATCTG  RP—CCAGGGCTGCGAGTCC  Reporter—CTGTGCGTCCGCCTTG |
| **Light Chain** | **Probe name** | **Sequences** |
| WT | Gm43291-1 WT | FP—GGATCGGAGAATAAGCATGAGTAGTT  RP—CCTCCAAATCTCCCACTTAAACGT  Reporter—CAGGTAGCGTGGTCTTCTAG |
| Leader | Igkj5-2 KO | FP—TGTCAGAGAAGCCCAAGCG  RP—GCCTGGTACCCAAGGGAGTA  Reporter—CTTCCACGCCTCTTTG |
| PGT121 | PGT121-Lam-1 KO | FP—ACAGACGGCCAGGATTACCT  RP—GCCTGGCTTCTGCTGGTA  Reporter—CTTCCAATGTTGTTTCCC |
| CLK09 | HuIgkv1-33 Tg | FP—GGAGACAGAGTCACCATCACTTG  RP—GCTTTCCCTGGTTTCTGCTGATA  Reporter—CAGGACATTAGCAACTATTTAA |
| CLK19 | CLK19-kappa-LC | FP—GCAGCCTGAAGATATTGCAACA  RP—CCACTTTGGTCCCAGGGC  Reporter—ACAGTATGCCACTTTCG |
| CLK21 | CLK21-kappa-LC | FP—GCGGAGGGACCAAGGT  RP—CCTCCAAATCTCCCACTTAAACGT  Reporter—TCACTTACGTTTTTTGATCTCC |

FP, Forward Primer; RP, Reverse Primer. Each mouse was genotyped with WT-, Leader- and the specific probes of each line. WT probes were used for the detection of WT allele; leader probes were used for the detection of the 5’end of the insertion; specific probes were used for the detection of pre-arranged VJ or VDJ insertion for each line respectively.

**Appendix Table S2. Genotyping result of PGT121κ KI mice**

| Line | TaqMan probes | | |
| --- | --- | --- | --- |
| PGT121κ | WT | PGT121LC | Leader |
| F0-1 | + | - | - |
| F0-2 | + | - | - |
| F0-3 | + | - | - |
| F0-4 | + | - | - |
| F0-5 | + | - | - |
| F0-6 | + | - | - |
| F0-7 | + | + | + |
| F0-8 | + | + | + |
| F0-9 | + | - | - |
| F0-10 | + | - | - |
| F0-11 | + | + | + |
| F0-12 | + | - | - |
| F0-13 | + | - | - |
| F0-14 | + | - | - |
| F0-15 | - | + | + |
| F0-16 | + | + | + |
| F0-17 | + | - | - |
| F0-18 | + | - | - |
| F0-19 | + | - | - |
| F0-20 | + | - | - |
| F0-21 | + | - | - |
| F0-22 | + | - | - |
| F0-23 | + | + | + |
| F0-24 | + | + | + |
| F0-25 | + | - | - |
| F0-26 | + | - | - |
| F0-27 | + | - | - |
| F0-28 | + | - | - |
| F0-29 | + | - | - |
| F0-30 | + | - | - |

“+”, positive signal; “-” negative signal.

**Appendix Table S3**. Genotyping result of CLK21 KI mice

| Line | TaqMan probes | | | | | |
| --- | --- | --- | --- | --- | --- | --- |
| CLK21 | WT | CLK21 HC | Leader | WT | CLK21 LC | Leader |
| F0-1 | + | - | - | + | + | + |
| F0-2 | + | + | + | + | + | + |
| F0-3 | + | + | + | + | + | + |
| F0-4 | + | - | - | + | + | + |
| F0-5 | + | - | - | + | - | - |
| F0-6 | + | + | + | + | + | + |
| F0-7 | + | + | + | + | - | - |
| F0-8 | + | - | - | + | - | - |
| F0-9 | + | + | + | + | - | - |
| F0-10 | + | - | - | + | - | - |
| F0-11 | + | - | - | + | - | - |
| F0-12 | + | + | + | + | + | + |
| F0-13 | + | - | - | + | - | - |
| F0-14 | + | + | + | + | - | - |

“+”, positive signal; “-” negative signal.

**Appendix Table S4. Genotyping result of CLK09 KI mice**

“+”, positive signal; “-” negative signal.

| Line | TaqMan probes | | | | | |
| --- | --- | --- | --- | --- | --- | --- |
| CLK09 | CLK09 HC | WT | Leader | CLK09 LC | WT | Leader |
| F0-1 | - | + | - | - | + | - |
| F0-2 | - | + | - | - | + | - |
| F0-3 | - | + | - | - | + | - |
| F0-4 | - | + | - | - | + | - |
| F0-5 | - | + | - | - | + | - |
| F0-6 | + | + | + | + | + | + |
| F0-7 | - | + | - | - | + | - |
| F0-8 | - | + | - | - | + | - |
| F0-9 | + | + | + | + | + | + |
| F0-10 | + | + | + | - | + | - |

**Appendix Table S5. Genotyping result of CLK19 KI mice**

| Line | TaqMan probes | | | | | |
| --- | --- | --- | --- | --- | --- | --- |
| CLK19 | CLK19 HC | WT | Leader | CLK19 LC | WT | Leader |
| F0-1 | + | + | + | - | + | - |
| F0-2 | UD4 | + | UD4 | UD4 | + | UD4 |
| F0-3 | - | + | - | - | + | - |
| F0-4 | - | + | - | - | + | - |
| F0-5 | + | + | + | - | + | - |
| F0-6 | - | + | - | - | + | - |
| F0-7 | - | + | - | - | + | - |
| F0-8 | + | + | + | - | + | - |
| F0-9 | + | + | + | + | + | + |
| F0-10 | - | + | - | - | + | - |
| F0-11 | - | + | - | - | + | - |
| F0-12 | + | + | + | + | + | + |
| F0-13 | - | + | - | - | + | - |
| F0-14 | - | + | - | - | + | - |
| F0-15 | + | + | + | - | + | - |
| F0-16 | - | + | - | - | + | - |
| F0-17 | - | + | - | - | + | - |
| F0-18 | - | + | - | - | + | - |
| F0-19 | - | + | - | - | + | - |
| F0-20 | - | + | - | - | + | - |
| F0-21 | - | + | - | - | + | UD4 |
| F0-22 | - | + | - | - | + | - |
| F0-23 | - | + | - | - | + | - |
| F0-24 | + | + | + | - | + | - |
| F0-25 | - | + | - | + | - | + |
| F0-26 | + | + | + | - | + | - |
| F0-27 | + | + | + | - | + | - |
| F0-28 | - | + | - | - | + | - |
| F0-29 | - | + | - | + | + | + |
| F0-30 | + | + | + | + | + | + |

“+”, positive signal; “-” negative signal, “UD4”, Signal between negative and positive ranges.

**Appendix Table S6 Primers of CLK for RT-PCR**

| **IgM primer** | | | |
| --- | --- | --- | --- |
|  | **Primer type** | **Primer name** | **Sequence** |
|  | FORWARD | CLK21HC-F | TGAGTGGATGGGATGGATCA |
|  | FORWARD | CLK09HC-F | CCGTGTATTACTGTGCGAGAG |
|  | FORWARD | CLK19HC-F | AGAAGTTTCAGGGCAGGGT |
|  | REVERSE | CLK21HC-R | GGGGAAGACATTTGGGAAGG |
|  | REVERSE | CLK09HC-R | GGGGAAGACATTTGGGAAGG |
|  | REVERSE | CLK19HC-R | GGGGAAGACATTTGGGAAGG |
| **IgKappa primer** | | | |
|  | FORWARD | CLK21LC-F | CTGCAGCCTGATGATTTTGC |
|  | FORWARD | CLK09LC-F | CAGCAGAAACCAGGGAAAGC |
|  | FORWARD | CLK19LC-F | CAGCAGAAACCAGGGAAAGC |
|  | REVERSE | CLK21LC-R | ACTGGATGGTGGGAAGATGG |
|  | REVERSE | CLK09LC-R | ACTGGATGGTGGGAAGATGG |
|  | REVERSE | CLK19LC-R | ACTGGATGGTGGGAAGATGG |

**Appendix Table S7 Index primers of NGS**

| **Primer Name** | **For/Rev/Probe** | **Description** | **Sequence** |
| --- | --- | --- | --- |
| JW65 | ATTACTCG | Illumina ID - D701 | CAAGCAGAAGACGGCATACGAGATCGAGTAATGTGACTGGAGTTCAGACGTGTGCTCTTCCGATCT |
| JW66 | TCCGGAGA | Illumina ID - D702 | CAAGCAGAAGACGGCATACGAGATTCTCCGGAGTGACTGGAGTTCAGACGTGTGCTCTTCCGATCT |
| JW67 | CGCTCATT | Illumina ID - D703 | CAAGCAGAAGACGGCATACGAGATAATGAGCGGTGACTGGAGTTCAGACGTGTGCTCTTCCGATCT |
| JW68 | GAGATTCC | Illumina ID - D704 | CAAGCAGAAGACGGCATACGAGATGGAATCTCGTGACTGGAGTTCAGACGTGTGCTCTTCCGATCT |
| JW69 | ATTCAGAA | Illumina ID - D705 | CAAGCAGAAGACGGCATACGAGATTTCTGAATGTGACTGGAGTTCAGACGTGTGCTCTTCCGATCT |
| JW70 | GAATTCGT | Illumina ID - D706 | CAAGCAGAAGACGGCATACGAGATACGAATTCGTGACTGGAGTTCAGACGTGTGCTCTTCCGATCT |
| JW71 | CTGAAGCT | Illumina ID - D707 | CAAGCAGAAGACGGCATACGAGATAGCTTCAGGTGACTGGAGTTCAGACGTGTGCTCTTCCGATCT |
| JW72 | TAATGCGC | Illumina ID - D708 | CAAGCAGAAGACGGCATACGAGATGCGCATTAGTGACTGGAGTTCAGACGTGTGCTCTTCCGATCT |
| JW73 | CGGCTATG | Illumina ID - D709 | CAAGCAGAAGACGGCATACGAGATCATAGCCGGTGACTGGAGTTCAGACGTGTGCTCTTCCGATCT |
| JW74 | TCCGCGAA | Illumina ID - D710 | CAAGCAGAAGACGGCATACGAGATTTCGCGGAGTGACTGGAGTTCAGACGTGTGCTCTTCCGATCT |
| JW75 | TCTCGCGC | Illumina ID - D711 | CAAGCAGAAGACGGCATACGAGATGCGCGAGAGTGACTGGAGTTCAGACGTGTGCTCTTCCGATCT |
| JW76 | AGCGATAG | Illumina ID - D712 | CAAGCAGAAGACGGCATACGAGATCTATCGCTGTGACTGGAGTTCAGACGTGTGCTCTTCCGATCT |
| JW77 | TATAGCCT | Illumina ID - D501 | AATGATACGGCGACCACCGAGATCTACACTATAGCCTACACTCTTTCCCTACACGACGCTCTTCCGATCT |
| JW78 | ATAGAGGC | Illumina ID - D502 | AATGATACGGCGACCACCGAGATCTACACATAGAGGCACACTCTTTCCCTACACGACGCTCTTCCGATCT |
| JW79 | CCTATCCT | Illumina ID - D503 | AATGATACGGCGACCACCGAGATCTACACCCTATCCTACACTCTTTCCCTACACGACGCTCTTCCGATCT |
| JW80 | GGCTCTGA | Illumina ID - D504 | AATGATACGGCGACCACCGAGATCTACACGGCTCTGAACACTCTTTCCCTACACGACGCTCTTCCGATCT |
| JW81 | AGGCGAAG | Illumina ID - D505 | AATGATACGGCGACCACCGAGATCTACACAGGCGAAGACACTCTTTCCCTACACGACGCTCTTCCGATCT |
| JW82 | TAATCTTA | Illumina ID - D506 | AATGATACGGCGACCACCGAGATCTACACTAATCTTAACACTCTTTCCCTACACGACGCTCTTCCGATCT |
| JW83 | CAGGACGT | Illumina ID - D507 | AATGATACGGCGACCACCGAGATCTACACCAGGACGTACACTCTTTCCCTACACGACGCTCTTCCGATCT |
| JW84 | GTACTGAC | Illumina ID - D508 | AATGATACGGCGACCACCGAGATCTACACGTACTGACACACTCTTTCCCTACACGACGCTCTTCCGATCT |
